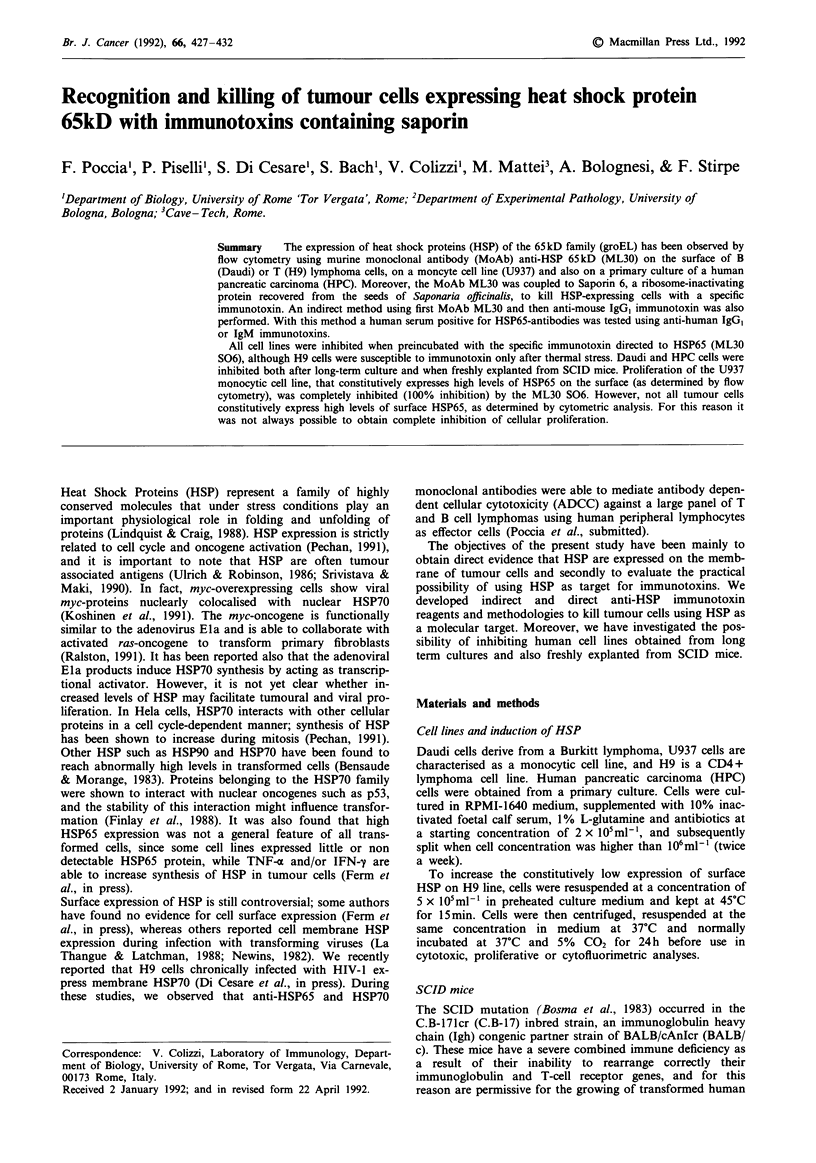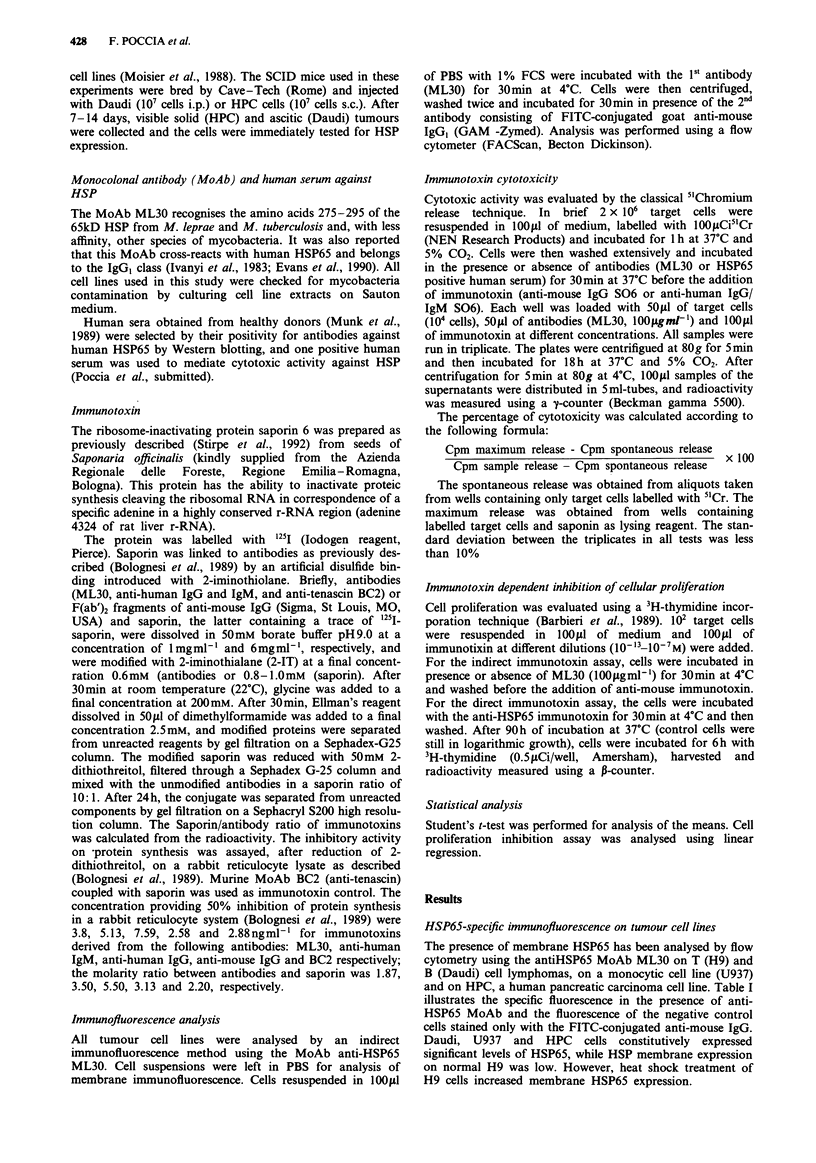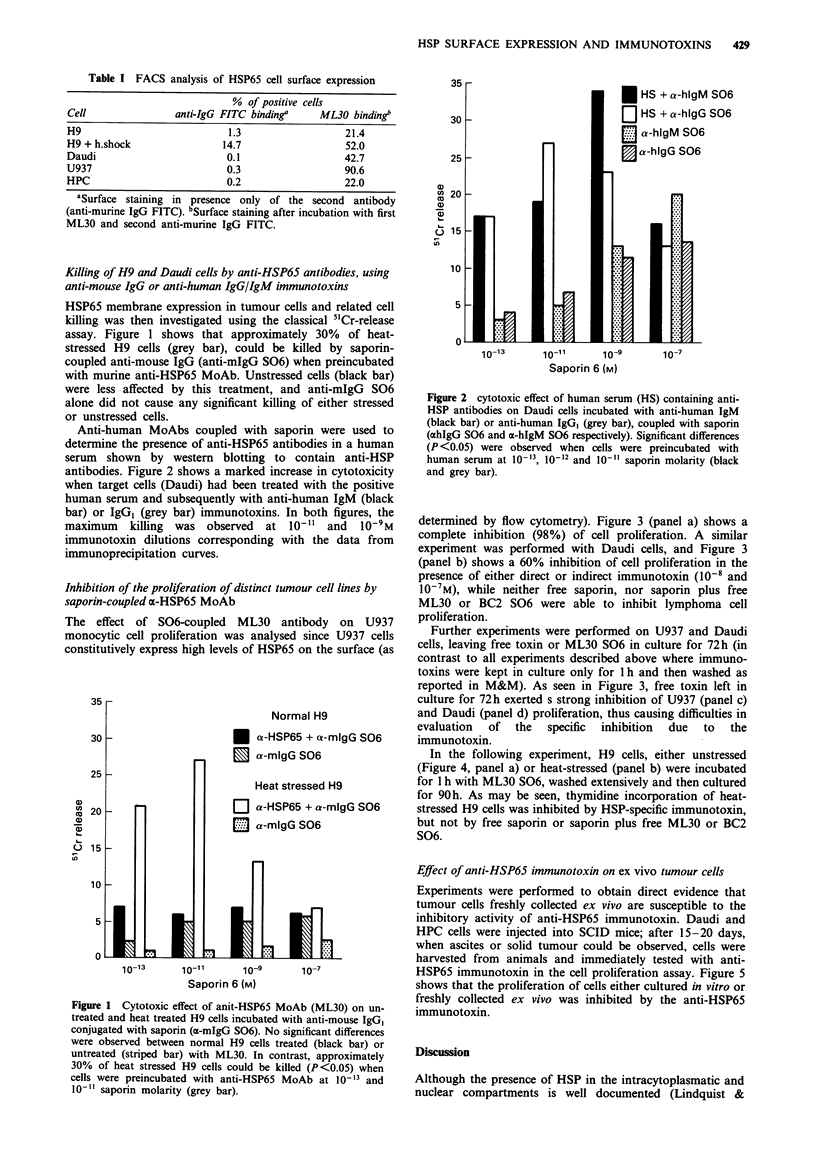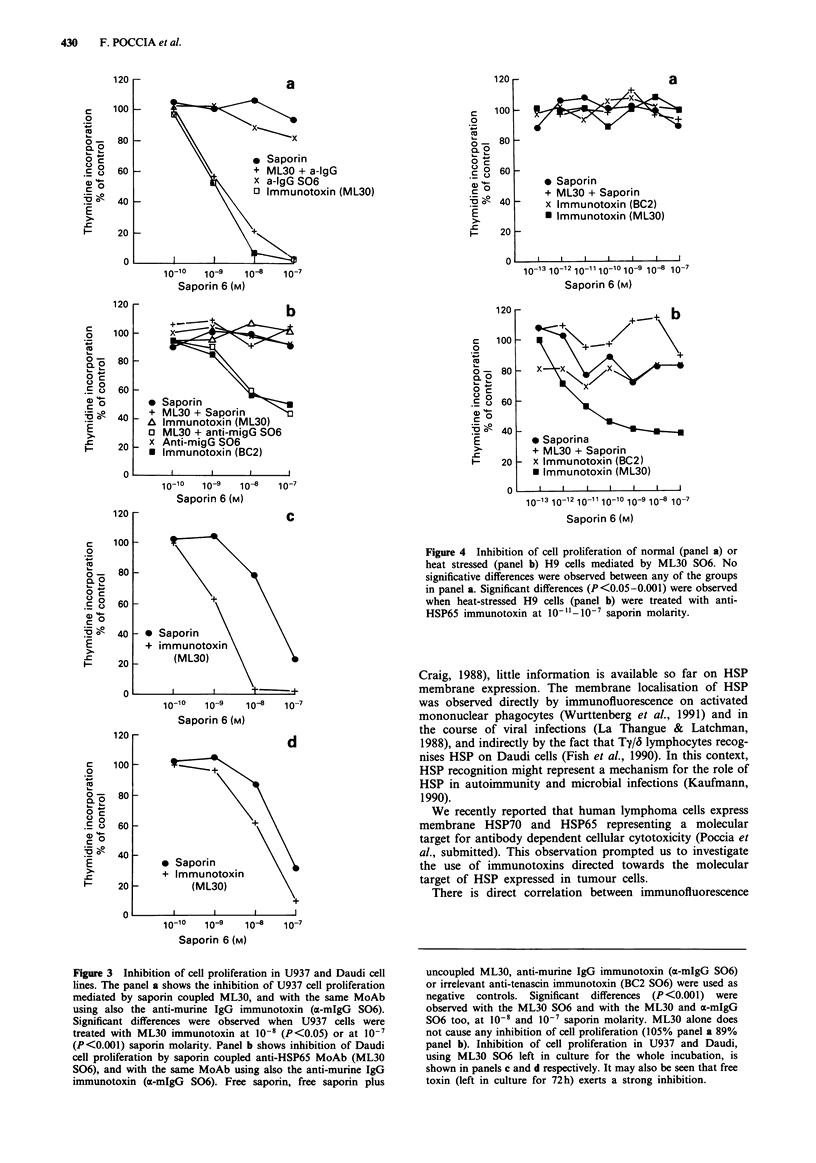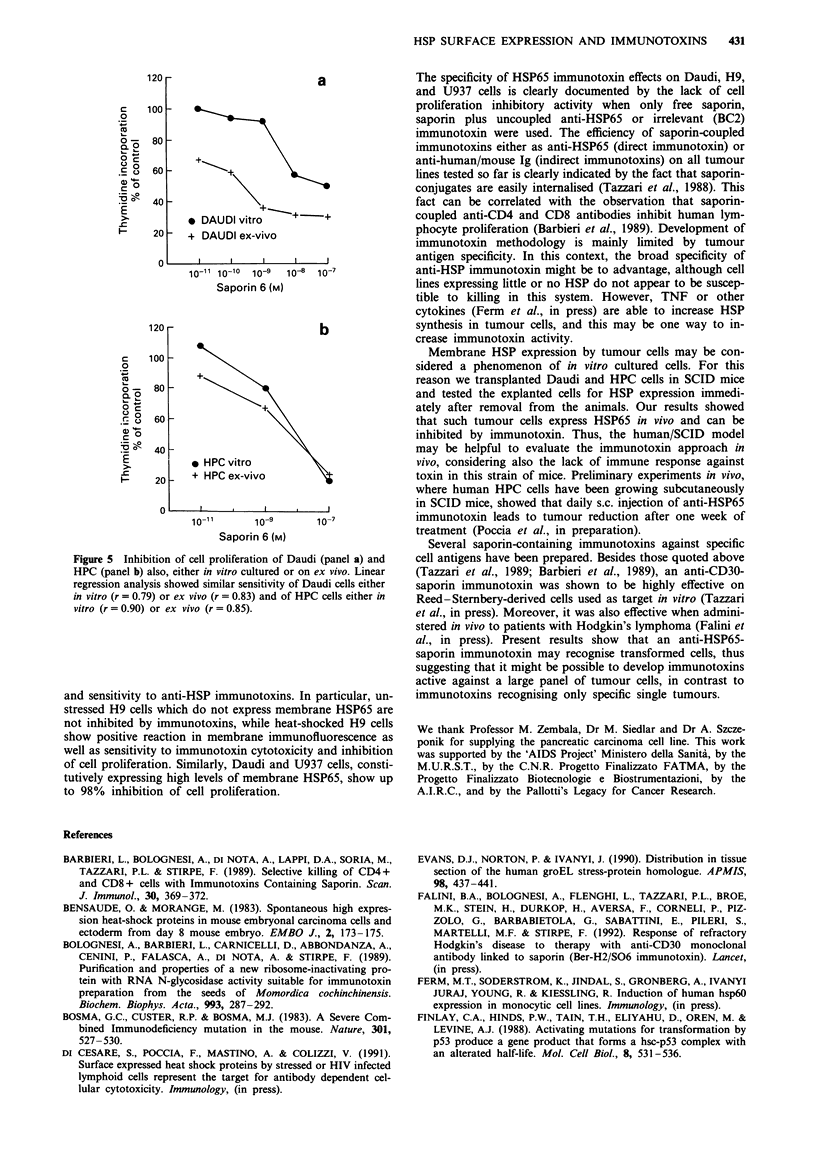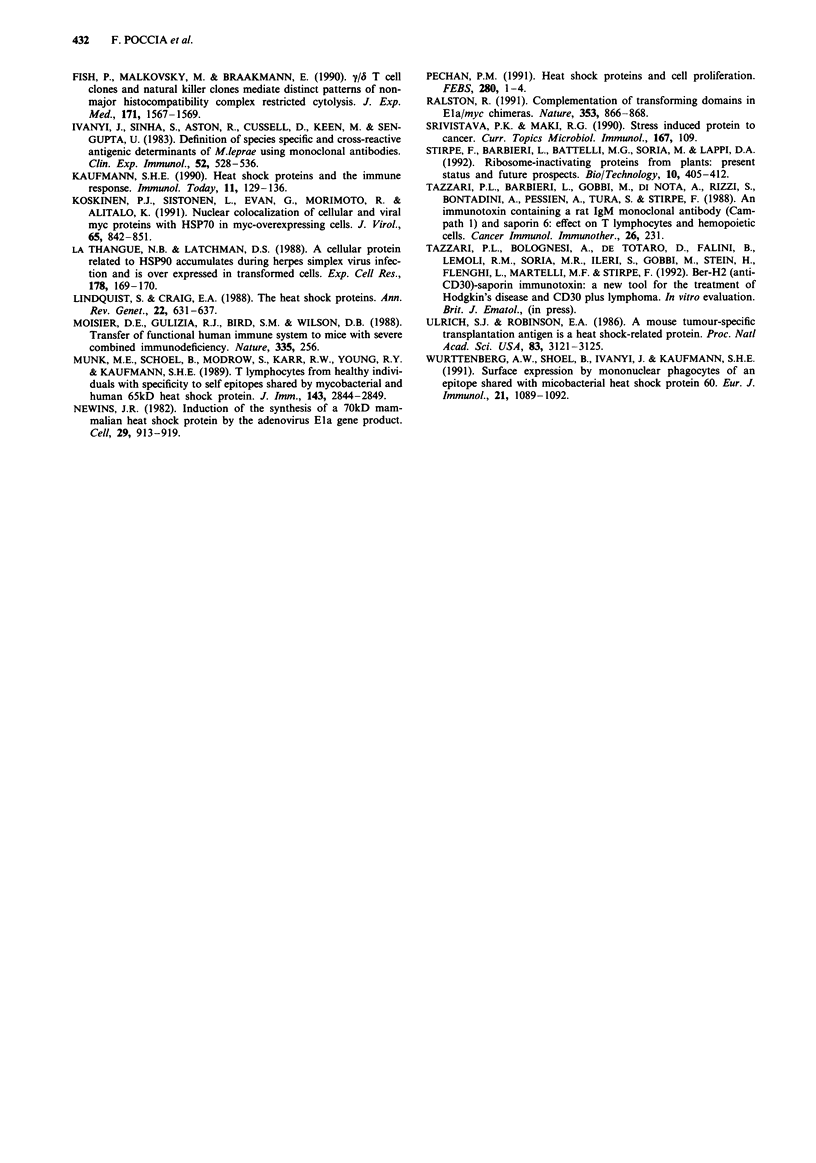# Recognition and killing of tumour cells expressing heat shock protein 65 kD with immunotoxins containing saporin.

**DOI:** 10.1038/bjc.1992.291

**Published:** 1992-09

**Authors:** F. Poccia, P. Piselli, S. Di Cesare, S. Bach, V. Colizzi, M. Mattei, A. Bolognesi, F. Stirpe

**Affiliations:** Department of Biology, University of Rome Tor Vergata, Italy.

## Abstract

The expression of heat shock proteins (HSP) of the 65 kD family (groEL) has been observed by flow cytometry using murine monoclonal antibody (MoAb) anti-HSP 65 kD (ML30) on the surface of B (Daudi) or T (H9) lymphoma cells, on a monocyte cell line (U937) and also on a primary culture of a human pancreatic carcinoma (HPC). Moreover, the MoAb ML30 was coupled to Saporin 6, a ribosome-inactivating protein recovered from the seeds of Saponaria officinalis, to kill HSP-expressing cells with a specific immunotoxin. An indirect method using first MoAb ML30 and then anti-mouse IgG1 immunotoxin was also performed. With this method a human serum positive for HSP65-antibodies was tested using anti-human IgG1 or IgM immunotoxins. All cell lines were inhibited when preincubated with the specific immunotoxin directed to HSP65 (ML30 SO6), although H9 cells were susceptible to immunotoxin only after thermal stress. Daudi and HPC cells were inhibited both after long-term culture and when freshly explanted from SCID mice. Proliferation of the U937 monocytic cell line, that constitutively expresses high levels of HSP65 on the surface (as determined by flow cytometry), was completely inhibited (100% inhibition) by the ML30 SO6. However, not all tumour cells constitutively express high levels of surface HSP65, as determined by cytometric analysis. For this reason it was not always possible to obtain complete inhibition of cellular proliferation.


Br. J. Cancer (1992), 66, 427–432 © Macmillan Press Ltd., 1992

### Recognition and killing of tumour cells expressing heat shock protein 65kD with immunotoxins containing saporin

F. Poccia[1], P. Piselli[1], S. Di Cesare[1], S. Bach[1], V. Colizzi[1], M. Mattei[3], A. Bolognesi, & F. Stirpe

[1]*Department of Biology, University of Rome 'Tor Vergata', Rome;* [2]*Department of Experimental Pathology, University of Bologna, Bologna;* [3]*Cave-Tech, Rome.*

**Summary** The expression of heat shock proteins (HSP) of the 65kD family (groEL) has been observed by flow cytometry using murine monoclonal antibody (MoAb) anti-HSP 65kD (ML30) on the surface of B (Daudi) or T (H9) lymphoma cells, on a moncyte cell line (U937) and also on a primary culture of a human pancreatic carcinoma (HPC). Moreover, the MoAb ML30 was coupled to Saporin 6, a ribosome-inactivating protein recovered from the seeds of *Saponaria officinalis*, to kill HSP-expressing cells with a specific immunotoxin. An indirect method using first MoAb ML30 and then anti-mouse $IgG_1$ immunotoxin was also performed. With this method a human serum positive for HSP65-antibodies was tested using anti-human $IgG_1$ or IgM immunotoxins.

All cell lines were inhibited when preincubated with the specific immunotoxin directed to HSP65 (ML30 SO6), although H9 cells were susceptible to immunotoxin only after thermal stress. Daudi and HPC cells were inhibited both after long-term culture and when freshly explanted from SCID mice. Proliferation of the U937 monocytic cell line, that constitutively expresses high levels of HSP65 on the surface (as determined by flow cytometry), was completely inhibited (100% inhibition) by the ML30 SO6. However, not all tumour cells constitutively express high levels of surface HSP65, as determined by cytometric analysis. For this reason it was not always possible to obtain complete inhibition of cellular proliferation.

Heat Shock Proteins (HSP) represent a family of highly conserved molecules that under stress conditions play an important physiological role in folding and unfolding of proteins (Lindquist & Craig, 1988). HSP expression is strictly related to cell cycle and oncogene activation (Pechan, 1991), and it is important to note that HSP are often tumour associated antigens (Ulrich & Robinson, 1986; Srivistava & Maki, 1990). In fact, *myc*-overexpressing cells show viral *myc*-proteins nuclearly colocalised with nuclear HSP70 (Koshinen *et al.*, 1991). The *myc*-oncogene is functionally similar to the adenovirus E1a and is able to collaborate with activated *ras*-oncogene to transform primary fibroblasts (Ralston, 1991). It has been reported also that the adenoviral E1a products induce HSP70 synthesis by acting as transcriptional activator. However, it is not yet clear whether increased levels of HSP may facilitate tumoural and viral proliferation. In Hela cells, HSP70 interacts with other cellular proteins in a cell cycle-dependent manner; synthesis of HSP has been shown to increase during mitosis (Pechan, 1991). Other HSP such as HSP90 and HSP70 have been found to reach abnormally high levels in transformed cells (Bensaude & Morange, 1983). Proteins belonging to the HSP70 family were shown to interact with nuclear oncogenes such as p53, and the stability of this interaction might influence transformation (Finlay *et al.*, 1988). It was also found that high HSP65 expression was not a general feature of all transformed cells, since some cell lines expressed little or non detectable HSP65 protein, while TNF-α and/or IFN-γ are able to increase synthesis of HSP in tumour cells (Ferm *et al.*, in press).

Surface expression of HSP is still controversial; some authors have found no evidence for cell surface expression (Ferm *et al.*, in press), whereas others reported cell membrane HSP expression during infection with transforming viruses (La Thangue & Latchman, 1988; Newins, 1982). We recently reported that H9 cells chronically infected with HIV-1 express membrane HSP70 (Di Cesare *et al.*, in press). During these studies, we observed that anti-HSP65 and HSP70 monoclonal antibodies were able to mediate antibody dependent cellular cytotoxicity (ADCC) against a large panel of T and B cell lymphomas using human peripheral lymphocytes as effector cells (Poccia *et al.*, submitted).

The objectives of the present study have been mainly to obtain direct evidence that HSP are expressed on the membrane of tumour cells and secondly to evaluate the practical possibility of using HSP as target for immunotoxins. We developed indirect and direct anti-HSP immunotoxin reagents and methodologies to kill tumour cells using HSP as a molecular target. Moreover, we have investigated the possibility of inhibiting human cell lines obtained from long term cultures and also freshly explanted from SCID mice.

### Materials and methods

### *Cell lines and induction of HSP*

Daudi cells derive from a Burkitt lymphoma, U937 cells are characterised as a monocytic cell line, and H9 is a CD4+ lymphoma cell line. Human pancreatic carcinoma (HPC) cells were obtained from a primary culture. Cells were cultured in RPMI-1640 medium, supplemented with 10% inactivated foetal calf serum, 1% L-glutamine and antibiotics at a starting concentration of $2 \times 10^5 ml^{-1}$, and subsequently split when cell concentration was higher than $10^6 ml^{-1}$ (twice a week).

To increase the constitutively low expression of surface HSP on H9 line, cells were resuspended at a concentration of $5 \times 10^5 ml^{-1}$ in preheated culture medium and kept at 45°C for 15min. Cells were then centrifuged, resuspended at the same concentration in medium at 37°C and normally incubated at 37°C and 5% $CO_2$ for 24h before use in cytotoxic, proliferative or cytofluorimetric analyses.

### *SCID mice*

The SCID mutation *(Bosma et al.,* 1983) occurred in the C.B-17Icr (C.B-17) inbred strain, an immunoglobulin heavy chain (Igh) congenic partner strain of BALB/cAnIcr (BALB/c). These mice have a severe combined immune deficiency as a result of their inability to rearrange correctly their immunoglobulin and T-cell receptor genes, and for this reason are permissive for the growing of transformed human

Correspondence: V. Colizzi, Laboratory of Immunology, Department of Biology, University of Rome, Tor Vergata, Via Carnevale, 00173 Rome, Italy.
Received 2 January 1992; and in revised form 22 April 1992.

cell lines (Moisier et al., 1988). The SCID mice used in these experiments were bred by Cave-Tech (Rome) and injected with Daudi ($10^7$ cells i.p.) or HPC cells ($10^7$ cells s.c.). After 7–14 days, visible solid (HPC) and ascitic (Daudi) tumours were collected and the cells were immediately tested for HSP expression.

*Monocolonal antibody (MoAb) and human serum against HSP*

The MoAb ML30 recognises the amino acids 275–295 of the 65kD HSP from *M. leprae* and *M. tuberculosis* and, with less affinity, other species of mycobacteria. It was also reported that this MoAb cross-reacts with human HSP65 and belongs to the $IgG_1$ class (Ivanyi et al., 1983; Evans et al., 1990). All cell lines used in this study were checked for mycobacteria contamination by culturing cell line extracts on Sauton medium.

Human sera obtained from healthy donors (Munk et al., 1989) were selected by their positivity for antibodies against human HSP65 by Western blotting, and one positive human serum was used to mediate cytotoxic activity against HSP (Poccia et al., submitted).

*Immunotoxin*

The ribosome-inactivating protein saporin 6 was prepared as previously described (Stirpe et al., 1992) from seeds of *Saponaria officinalis* (kindly supplied from the Azienda Regionale delle Foreste, Regione Emilia-Romagna, Bologna). This protein has the ability to inactivate proteic synthesis cleaving the ribosomal RNA in correspondence of a specific adenine in a highly conserved r-RNA region (adenine 4324 of rat liver r-RNA).

The protein was labelled with $^{125}I$ (Iodogen reagent, Pierce). Saporin was linked to antibodies as previously described (Bolognesi et al., 1989) by an artificial disulfide binding introduced with 2-iminothiolane. Briefly, antibodies (ML30, anti-human IgG and IgM, and anti-tenascin BC2) or $F(ab')_2$ fragments of anti-mouse IgG (Sigma, St Louis, MO, USA) and saporin, the latter containing a trace of $^{125}I$-saporin, were dissolved in 50mM borate buffer pH9.0 at a concentration of $1\,mg\,ml^{-1}$ and $6\,mg\,ml^{-1}$, respectively, and were modified with 2-iminothialane (2-IT) at a final concentration 0.6mM (antibodies or 0.8–1.0mM (saporin). After 30min at room temperature (22°C), glycine was added to a final concentration at 200mM. After 30min, Ellman's reagent dissolved in 50µl of dimethylformamide was added to a final concentration 2.5mM, and modified proteins were separated from unreacted reagents by gel filtration on a Sephadex-G25 column. The modified saporin was reduced with 50mM 2-dithiothreitol, filtered through a Sephadex G-25 column and mixed with the unmodified antibodies in a saporin ratio of 10:1. After 24h, the conjugate was separated from unreacted components by gel filtration on a Sephacryl S200 high resolution column. The Saporin/antibody ratio of immunotoxins was calculated from the radioactivity. The inhibitory activity on protein synthesis was assayed, after reduction of 2-dithiothreitol, on a rabbit reticulocyte lysate as described (Bolognesi et al., 1989). Murine MoAb BC2 (anti-tenascin) coupled with saporin was used as immunotoxin control. The concentration providing 50% inhibition of protein synthesis in a rabbit reticulocyte system (Bolognesi et al., 1989) were 3.8, 5.13, 7.59, 2.58 and $2.88\,ng\,ml^{-1}$ for immunotoxins derived from the following antibodies: ML30, anti-human IgM, anti-human IgG, anti-mouse IgG and BC2 respectively; the molarity ratio between antibodies and saporin was 1.87, 3.50, 5.50, 3.13 and 2.20, respectively.

*Immunofluorescence analysis*

All tumour cell lines were analysed by an indirect immunofluorescence method using the MoAb anti-HSP65 ML30. Cell suspensions were left in PBS for analysis of membrane immunofluorescence. Cells resuspended in 100µl of PBS with 1% FCS were incubated with the 1st antibody (ML30) for 30min at 4°C. Cells were then centrifuged, washed twice and incubated for 30min in presence of the 2nd antibody consisting of FITC-conjugated goat anti-mouse $IgG_1$ (GAM -Zymed). Analysis was performed using a flow cytometer (FACScan, Becton Dickinson).

*Immunotoxin cytotoxicity*

Cytotoxic activity was evaluated by the classical $^{51}$Chromium release technique. In brief $2 \times 10^6$ target cells were resuspended in 100µl of medium, labelled with 100µCi$^{51}$Cr (NEN Research Products) and incubated for 1h at 37°C and 5% $CO_2$. Cells were then washed extensively and incubated in the presence or absence of antibodies (ML30 or HSP65 positive human serum) for 30min at 37°C before the addition of immunotoxin (anti-mouse IgG SO6 or anti-human IgG/IgM SO6). Each well was loaded with 50µl of target cells ($10^4$ cells), 50µl of antibodies (ML30, $100\,\mu g\,ml^{-1}$) and 100µl of immunotoxin at different concentrations. All samples were run in triplicate. The plates were centrifigued at 80g for 5min and then incubated for 18h at 37°C and 5% $CO_2$. After centrifugation for 5min at 80g at 4°C, 100µl samples of the supernatants were distributed in 5ml-tubes, and radioactivity was measured using a γ-counter (Beckman gamma 5500).

The percentage of cytotoxicity was calculated according to the following formula:

$$\frac{\text{Cpm maximum release - Cpm spontaneous release}}{\text{Cpm sample release} - \text{Cpm spontaneous release}} \times 100$$

The spontaneous release was obtained from aliquots taken from wells containing only target cells labelled with $^{51}Cr$. The maximum release was obtained from wells containing labelled target cells and saponin as lysing reagent. The standard deviation between the triplicates in all tests was less than 10%

*Immunotoxin dependent inhibition of cellular proliferation*

Cell proliferation was evaluated using a $^3$H-thymidine incorporation technique (Barbieri et al., 1989). $10^2$ target cells were resuspended in 100µl of medium and 100µl of immunotixin at different dilutions ($10^{-13}$–$10^{-7}$M) were added. For the indirect immunotoxin assay, cells were incubated in presence or absence of ML30 ($100\,\mu g\,ml^{-1}$) for 30min at 4°C and washed before the addition of anti-mouse immunotoxin. For the direct immunotoxin assay, the cells were incubated with the anti-HSP65 immunotoxin for 30min at 4°C and then washed. After 90h of incubation at 37°C (control cells were still in logarithmic growth), cells were incubated for 6h with $^3$H-thymidine (0.5µCi/well, Amersham), harvested and radioactivity measured using a β-counter.

*Statistical analysis*

Student's *t*-test was performed for analysis of the means. Cell proliferation inhibition assay was analysed using linear regression.

### Results

*HSP65-specific immunofluorescence on tumour cell lines*

The presence of membrane HSP65 has been analysed by flow cytometry using the antiHSP65 MoAb ML30 on T (H9) and B (Daudi) cell lymphomas, on a monocytic cell line (U937) and on HPC, a human pancreatic carcinoma cell line. Table I illustrates the specific fluorescence in the presence of anti-HSP65 MoAb and the fluorescence of the negative control cells stained only with the FITC-conjugated anti-mouse IgG. Daudi, U937 and HPC cells constitutively expressed significant levels of HSP65, while HSP membrane expression on normal H9 was low. However, heat shock treatment of H9 cells increased membrane HSP65 expression.

**Table I** FACS analysis of HSP65 cell surface expression

| Cell | % of positive cells anti-IgG FITC binding[a] | ML30 binding[b] |
|---|---|---|
| H9 | 1.3 | 21.4 |
| H9 + h.shock | 14.7 | 52.0 |
| Daudi | 0.1 | 42.7 |
| U937 | 0.3 | 90.6 |
| HPC | 0.2 | 22.0 |

[a]Surface staining in presence only of the second antibody (anti-murine IgG FITC). [b]Surface staining after incubation with first ML30 and second anti-murine IgG FITC.

*Killing of H9 and Daudi cells by anti-HSP65 antibodies, using anti-mouse IgG or anti-human IgG/IgM immunotoxins*

HSP65 membrane expression in tumour cells and related cell killing was then investigated using the classical $^{51}$Cr-release assay. Figure 1 shows that approximately 30% of heat-stressed H9 cells (grey bar), could be killed by saporin-coupled anti-mouse IgG (anti-mIgG SO6) when preincubated with murine anti-HSP65 MoAb. Unstressed cells (black bar) were less affected by this treatment, and anti-mIgG SO6 alone did not cause any significant killing of either stressed or unstressed cells.

Anti-human MoAbs coupled with saporin were used to determine the presence of anti-HSP65 antibodies in a human serum shown by western blotting to contain anti-HSP antibodies. Figure 2 shows a marked increase in cytotoxicity when target cells (Daudi) had been treated with the positive human serum and subsequently with anti-human IgM (black bar) or $IgG_1$ (grey bar) immunotoxins. In both figures, the maximum killing was observed at $10^{-11}$ and $10^{-9}$M immunotoxin dilutions corresponding with the data from immunoprecipitation curves.

*Inhibition of the proliferation of distinct tumour cell lines by saporin-coupled* α-HSP65 MoAb

The effect of SO6-coupled ML30 antibody on U937 monocytic cell proliferation was analysed since U937 cells constitutively express high levels of HSP65 on the surface (as determined by flow cytometry). Figure 3 (panel a) shows a complete inhibition (98%) of cell proliferation. A similar experiment was performed with Daudi cells, and Figure 3 (panel b) shows a 60% inhibition of cell proliferation in the presence of either direct or indirect immunotoxin ($10^{-8}$ and $10^{-7}$M), while neither free saporin, nor saporin plus free ML30 or BC2 SO6 were able to inhibit lymphoma cell proliferation.

Further experiments were performed on U937 and Daudi cells, leaving free toxin or ML30 SO6 in culture for 72h (in contrast to all experiments described above where immunotoxins were kept in culture only for 1h and then washed as reported in M&M). As seen in Figure 3, free toxin left in culture for 72h exerted s strong inhibition of U937 (panel c) and Daudi (panel d) proliferation, thus causing difficulties in evaluation of the specific inhibition due to the immunotoxin.

In the following experiment, H9 cells, either unstressed (Figure 4, panel a) or heat-stressed (panel b) were incubated for 1h with ML30 SO6, washed extensively and then cultured for 90h. As may be seen, thymidine incorporation of heat-stressed H9 cells was inhibited by HSP-specific immunotoxin, but not by free saporin or saporin plus free ML30 or BC2 SO6.

**Figure 1** Cytotoxic effect of anit-HSP65 MoAb (ML30) on untreated and heat treated H9 cells incubated with anti-mouse $IgG_1$ conjugated with saporin (α-mIgG SO6). No significant differences were observed between normal H9 cells treated (black bar) or untreated (striped bar) with ML30. In contrast, approximately 30% of heat stressed H9 cells could be killed ($P<0.05$) when cells were preincubated with anti-HSP65 MoAb at $10^{-13}$ and $10^{-11}$ saporin molarity (grey bar).

**Figure 2** cytotoxic effect of human serum (HS) containing anti-HSP antibodies on Daudi cells incubated with anti-human IgM (black bar) or anti-human $IgG_1$ (grey bar), coupled with saporin (αhIgG SO6 and α-hIgM SO6 respectively). Significant differences ($P<0.05$) were observed when cells were preincubated with human serum at $10^{-13}$, $10^{-12}$ and $10^{-11}$ saporin molarity (black and grey bar).

*Effect of anti-HSP65 immunotoxin on* ex vivo *tumour cells*

Experiments were performed to obtain direct evidence that tumour cells freshly collected *ex vivo* are susceptible to the inhibitory activity of anti-HSP65 immunotoxin. Daudi and HPC cells were injected into SCID mice; after 15–20 days, when ascites or solid tumour could be observed, cells were harvested from animals and immediately tested with anti-HSP65 immunotoxin in the cell proliferation assay. Figure 5 shows that the proliferation of cells either cultured *in vitro* or freshly collected *ex vivo* was inhibited by the anti-HSP65 immunotoxin.

### Discussion

Although the presence of HSP in the intracytoplasmatic and nuclear compartments is well documented (Lindquist &

Figure 3 Inhibition of cell proliferation in U937 and Daudi cell lines. The panel **a** shows the inhibition of U937 cell proliferation mediated by saporin coupled ML30, and with the same MoAb using also the anti-murine IgG immunotoxin (α-mIgG SO6). Significant differences were observed when U937 cells were treated with ML30 immunotoxin at $10^{-8}$ ($P<0.05$) or at $10^{-7}$ ($P<0.001$) saporin molarity. Panel **b** shows inhibition of Daudi cell proliferation by saporin coupled anti-HSP65 MoAb (ML30 SO6), and with the same MoAb using also the anti-murine IgG immunotoxin (α-mIgG SO6). Free saporin, free saporin plus uncoupled ML30, anti-murine IgG immunotoxin (α-mIgG SO6) or irrelevant anti-tenascin immunotoxin (BC2 SO6) were used as negative controls. Significant differences ($P<0.001$) were observed with the ML30 SO6 and with the ML30 and α-mIgG SO6 too, at $10^{-8}$ and $10^{-7}$ saporin molarity. ML30 alone does not cause any inhibition of cell proliferation (105% panel **a** 89% panel **b**). Inhibition of cell proliferation in U937 and Daudi, using ML30 SO6 left in culture for the whole incubation, is shown in panels **c** and **d** respectively. It may also be seen that free toxin (left in culture for 72h) exerts a strong inhibition.

**Figure 4** Inhibition of cell proliferation of normal (panel **a**) or heat stressed (panel **b**) H9 cells mediated by ML30 SO6. No significative differences were observed between any of the groups in panel **a**. Significant differences ($P<0.05-0.001$) were observed when heat-stressed H9 cells (panel **b**) were treated with anti-HSP65 immunotoxin at $10^{-11}-10^{-7}$ saporin molarity.

Craig, 1988), little information is available so far on HSP membrane expression. The membrane localisation of HSP was observed directly by immunofluorescence on activated mononuclear phagocytes (Wurttenberg *et al.*, 1991) and in the course of viral infections (La Thangue & Latchman, 1988), and indirectly by the fact that Tγ/δ lymphocytes recognises HSP on Daudi cells (Fish *et al.*, 1990). In this context, HSP recognition might represent a mechanism for the role of HSP in autoimmunity and microbial infections (Kaufmann, 1990).

We recently reported that human lymphoma cells express membrane HSP70 and HSP65 representing a molecular target for antibody dependent cellular cytotoxicity (Poccia *et al.*, submitted). This observation prompted us to investigate the use of immunotoxins directed towards the molecular target of HSP expressed in tumour cells.

There is direct correlation between immunofluorescence

**Figure 5** Inhibition of cell proliferation of Daudi (panel **a**) and HPC (panel **b**) also, either *in vitro* cultured or on *ex vivo*. Linear regression analysis showed similar sensitivity of Daudi cells either *in vitro* ($r = 0.79$) or *ex vivo* ($r = 0.83$) and of HPC cells either *in vitro* ($r = 0.90$) or *ex vivo* ($r = 0.85$).

and sensitivity to anti-HSP immunotoxins. In particular, unstressed H9 cells which do not express membrane HSP65 are not inhibited by immunotoxins, while heat-shocked H9 cells show positive reaction in membrane immunofluorescence as well as sensitivity to immunotoxin cytotoxicity and inhibition of cell proliferation. Similarly, Daudi and U937 cells, constitutively expressing high levels of membrane HSP65, show up to 98% inhibition of cell proliferation.

The specificity of HSP65 immunotoxin effects on Daudi, H9, and U937 cells is clearly documented by the lack of cell proliferation inhibitory activity when only free saporin, saporin plus uncoupled anti-HSP65 or irrelevant (BC2) immunotoxin were used. The efficiency of saporin-coupled immunotoxins either as anti-HSP65 (direct immunotoxin) or anti-human/mouse Ig (indirect immunotoxins) on all tumour lines tested so far is clearly indicated by the fact that saporin-conjugates are easily internalised (Tazzari *et al.*, 1988). This fact can be correlated with the observation that saporin-coupled anti-CD4 and CD8 antibodies inhibit human lymphocyte proliferation (Barbieri *et al.*, 1989). Development of immunotoxin methodology is mainly limited by tumour antigen specificity. In this context, the broad specificity of anti-HSP immunotoxin might be to advantage, although cell lines expressing little or no HSP do not appear to be susceptible to killing in this system. However, TNF or other cytokines (Ferm *et al.*, in press) are able to increase HSP synthesis in tumour cells, and this may be one way to increase immunotoxin activity.

Membrane HSP expression by tumour cells may be considered a phenomenon of *in vitro* cultured cells. For this reason we transplanted Daudi and HPC cells in SCID mice and tested the explanted cells for HSP expression immediately after removal from the animals. Our results showed that such tumour cells express HSP65 *in vivo* and can be inhibited by immunotoxin. Thus, the human/SCID model may be helpful to evaluate the immunotoxin approach *in vivo*, considering also the lack of immune response against toxin in this strain of mice. Preliminary experiments *in vivo*, where human HPC cells have been growing subcutaneously in SCID mice, showed that daily s.c. injection of anti-HSP65 immunotoxin leads to tumour reduction after one week of treatment (Poccia *et al.*, in preparation).

Several saporin-containing immunotoxins against specific cell antigens have been prepared. Besides those quoted above (Tazzari *et al.*, 1989; Barbieri *et al.*, 1989), an anti-CD30-saporin immunotoxin was shown to be highly effective on Reed–Sternbery-derived cells used as target *in vitro* (Tazzari *et al.*, in press). Moreover, it was also effective when administered *in vivo* to patients with Hodgkin's lymphoma (Falini *et al.*, in press). Present results show that an anti-HSP65-saporin immunotoxin may recognise transformed cells, thus suggesting that it might be possible to develop immunotoxins active against a large panel of tumour cells, in contrast to immunotoxins recognising only specific single tumours.

We thank Professor M. Zembala, Dr M. Siedlar and Dr A. Szczeponik for supplying the pancreatic carcinoma cell line. This work was supported by the 'AIDS Project' Ministero della Sanità, by the M.U.R.S.T., by the C.N.R. Progetto Finalizzato FATMA, by the Progetto Finalizzato Biotecnologie e Biostrumentazioni, by the A.I.R.C., and by the Pallotti's Legacy for Cancer Research.

### References

BARBIERI, L., BOLOGNESI, A., DI NOTA, A., LAPPI, D.A., SORIA, M., TAZZARI, P.L. & STIRPE, F. (1989). Selective killing of CD4+ and CD8+ cells with Immunotoxins Containing Saporin. *Scan. J. Immunol.*, **30**, 369–372.

BENSAUDE, O. & MORANGE, M. (1983). Spontaneous high expression heat-shock proteins in mouse embryonal carcinoma cells and ectoderm from day 8 mouse embryo. *EMBO J.*, **2**, 173–175.

BOLOGNESI, A., BARBIERI, L., CARNICELLI, D., ABBONDANZA, A., CENINI, P., FALASCA, A., DI NOTA, A. & STIRPE, F. (1989). Purification and properties of a new ribosome-inactivating protein with RNA N-glycosidase activity suitable for immunotoxin preparation from the seeds of *Momordica cochinchinensis*. *Biochem. Biophys. Acta.*, **993**, 287–292.

BOSMA, G.C., CUSTER, R.P. & BOSMA, M.J. (1983). A Severe Combined Immunodeficiency mutation in the mouse. *Nature*, **301**, 527–530.

DI CESARE, S., POCCIA, F., MASTINO, A. & COLIZZI, V. (1991). Surface expressed heat shock proteins by stressed or HIV infected lymphoid cells represent the target for antibody dependent cellular cytotoxicity. *Immunology*, (in press).

EVANS, D.J., NORTON, P. & IVANYI, J. (1990). Distribution in tissue section of the human groEL stress-protein homologue. *APMIS*, **98**, 437–441.

FALINI, B.A., BOLOGNESI, A., FLENGHI, L., TAZZARI, P.L., BROE, M.K., STEIN, H., DURKOP, H., AVERSA, F., CORNELI, P., PIZZOLO, G., BARBABIETOLA, G., SABATTINI, E., PILERI, S., MARTELLI, M.F. & STIRPE, F. (1992). Response of refractory Hodgkin's disease to therapy with anti-CD30 monoclonal antibody linked to saporin (Ber-H2/SO6 immunotoxin). *Lancet*, (in press).

FERM, M.T., SODERSTROM, K., JINDAL, S., GRONBERG, A., IVANYI JURAJ, YOUNG, R. & KIESSLING, R. Induction of human hsp60 expression in monocytic cell lines. *Immunology*, (in press).

FINLAY, C.A., HINDS, P.W., TAIN, T.H., ELIYAHU, D., OREN, M. & LEVINE, A.J. (1988). Activating mutations for transformation by p53 produce a gene product that forms a hsc-p53 complex with an alterated half-life. *Mol. Cell Biol.*, **8**, 531–536.

FISH, P., MALKOVSKY, M. & BRAAKMANN, E. (1990). $\gamma/\delta$ T cell clones and natural killer clones mediate distinct patterns of non-major histocompatibility complex restricted cytolysis. *J. Exp. Med.*, **171**, 1567–1569.

IVANYI, J., SINHA, S., ASTON, R., CUSSELL, D., KEEN, M. & SENGUPTA, U. (1983). Definition of species specific and cross-reactive antigenic determinants of *M.leprae* using monoclonal antibodies. *Clin. Exp. Immunol.*, **52**, 528–536.

KAUFMANN, S.H.E. (1990). Heat shock proteins and the immune response. *Immunol. Today*, **11**, 129–136.

KOSKINEN, P.J., SISTONEN, L., EVAN, G., MORIMOTO, R. & ALITALO, K. (1991). Nuclear colocalization of cellular and viral myc proteins with HSP70 in myc-overexpressing cells. *J. Virol.*, **65**, 842–851.

LA THANGUE, N.B. & LATCHMAN, D.S. (1988). A cellular protein related to HSP90 accumulates during herpes simplex virus infection and is over expressed in transformed cells. *Exp. Cell Res.*, **178**, 169–170.

LINDQUIST, S. & CRAIG, E.A. (1988). The heat shock proteins. *Ann. Rev. Genet.*, **22**, 631–637.

MOISIER, D.E., GULIZIA, R.J., BIRD, S.M. & WILSON, D.B. (1988). Transfer of functional human immune system to mice with severe combined immunodeficiency. *Nature*, **335**, 256.

MUNK, M.E., SCHOEL, B., MODROW, S., KARR, R.W., YOUNG, R.Y. & KAUFMANN, S.H.E. (1989). T lymphocytes from healthy individuals with specificity to self epitopes shared by mycobacterial and human 65kD heat shock protein. *J. Imm.*, **143**, 2844–2849.

NEWINS, J.R. (1982). Induction of the synthesis of a 70kD mammalian heat shock protein by the adenovirus Ela gene product. *Cell*, **29**, 913–919.

PECHAN, P.M. (1991). Heat shock proteins and cell proliferation. *FEBS*, **280**, 1–4.

RALSTON, R. (1991). Complementation of transforming domains in Ela/*myc* chimeras. *Nature*, **353**, 866–868.

SRIVISTAVA, P.K. & MAKI, R.G. (1990). Stress induced protein to cancer. *Curr. Topics Microbiol. Immunol.*, **167**, 109.

STIRPE, F., BARBIERI, L., BATTELLI, M.G., SORIA, M. & LAPPI, D.A. (1992). Ribosome-inactivating proteins from plants: present status and future prospects. *Bio/Technology*, **10**, 405–412.

TAZZARI, P.L., BARBIERI, L., GOBBI, M., DI NOTA, A., RIZZI, S., BONTADINI, A., PESSIEN, A., TURA, S. & STIRPE, F. (1988). An immunotoxin containing a rat IgM monoclonal antibody (Campath 1) and saporin 6: effect on T lymphocytes and hemopoietic cells. *Cancer Immunol. Immunother.*, **26**, 231.

TAZZARI, P.L., BOLOGNESI, A., DE TOTARO, D., FALINI, B., LEMOLI, R.M., SORIA, M.R., ILERI, S., GOBBI, M., STEIN, H., FLENGHI, L., MARTELLI, M.F. & STIRPE, F. (1992). Ber-H2 (anti-CD30)-saporin immunotoxin: a new tool for the treatment of Hodgkin's disease and CD30 plus lymphoma. *In vitro* evaluation. *Brit. J. Ematol.*, (in press).

ULRICH, S.J. & ROBINSON, E.A. (1986). A mouse tumour-specific transplantation antigen is a heat shock-related protein. *Proc. Natl Acad. Sci. USA*, **83**, 3121–3125.

WURTTENBERG, A.W., SHOEL, B., IVANYI, J. & KAUFMANN, S.H.E. (1991). Surface expression by mononuclear phagocytes of an epitope shared with micobacterial heat shock protein 60. *Eur. J. Immunol.*, **21**, 1089–1092.